# A Bibliometric Evaluation of the Top 100 Cited Dimethyl Fumarate Articles

**DOI:** 10.3390/molecules26041085

**Published:** 2021-02-19

**Authors:** Francisco Javier García-Fernández, Alba Estela García-Fernández, Ichiro Ikuta, Eduardo Nava, Julian Solis García del Pozo, Joaquin Jordan, Maria F. Galindo

**Affiliations:** 1 Servicio de Análisis Clínicos, Hospital General de Tomelloso, 13700 Tomelloso, Spain; jgardez@hotmail.com; 2 Bioquímica Clínica, Hospital Vall d’Hebron, 08023 Barcelona, Spain; asgfernandez@gmail.com; 3 Neuroradiology Section, Yale Center for Imaging Informatics, Department of Radiology & Biomedical Imaging, Yale University School of Medicine, New Haven, CT 065610, USA; ichiroikuta@hotmail.com; 4 Departamento de Ciencias Médicas, Facultad de Medicina de Albacete Universidad Castilla La Mancha, GAI, 02008 Albacete, Spain; eduardo.nava@uclm.es (E.N.); julianeloysolis@gmail.com (J.S.G.d.P.); joaquin.jordan@uclm.es (J.J.); 5 Servicio de Medicina Interna, Complejo Hospitalario Universitario de Albacete, GAI, 02006 Albacete, Spain; 6 Departamento de Ciencias Médicas, Facultad de Farmacia, Universidad Castilla La Mancha, 02008 Albacete, Spain

**Keywords:** dimethyl fumarate, Tecfidera, multiple sclerosis, bibliometric study

## Abstract

Dimethyl fumarate is a cytoprotective and immunomodulatory drug used in the treatment of multiple sclerosis. We performed a bibliometric study examining the characteristics and trends of the top 100 cited articles that include dimethyl fumarate in the title. On 21 September 2020 we carried out an electronic search in the Web of Science (WOS), seeking articles that include the following terms within the title: dimethyl fumarate, BG-12, or Tecfidera. To focus our investigation on original research, we refined the search to include only articles, early access, others, case report, and clinical trials. We obtained a total of 1115 items, which were cited 7169 times, had a citation density of 6.43 citations/item, and an h-index of 40. Around 2010, there was a jump in the number of published articles per year, rising from 5 articles/year up to 12 articles/year. We sorted all the items by the number of citations and selected the top 100 most cited (T100). The T100 had 4164 citations, with a density of 37 citations/year and contained 16 classic research articles. They were published between 1961 and 2018; the years 2010–2018 amassed nearly 80% of the T100. We noted 17 research areas with articles in the T100. Of these, the number one ranking went to neurosciences/neurology with 39 articles, and chemistry ranked second on the T100 list with 14 items. We noticed that the percentage of articles belonging to different journals changed depending on the time period. Chemistry held the highest number of papers during 1961–2000, while pharmacology andneurosciences/neurology led the 2001–2018 interval. A total of 478 authors from 145 institutions and 25 countries were included in the T100 ranking. The paper by Gold R et al. was the most successful with 14 articles, 1.823 citations and a density of 140.23 citations/year. The biotechnological company Biogen led the T100 list with 20 articles. With 59 published articles, the USA was the leading country in publications. We concluded that this study analyzed the use of and research on dimethyl fumarate from a different perspective, which will allow the readership (expert or not) to understand the relevance of classic and recent literature on this topic.

## 1. Introduction

Among the many difficulties researchers encounter when facing a research topic, one is the huge number of journals and articles that are periodically published. It is often hard to understand or grasp the relevance of a particular article, especially when the article is not recent. In a totally subjective manner, the choice to read an article and interpret its relevance to the research topic may be left to a reader’s intuition and biases. Bibliometric analysis is considered a very useful tool to overcome this problem, since it offers a cross-sectional view as well as the current state of research work on the topic of interest. Usually, a bibliometric analysis aims to identify the academic impact and features of a number of publications within a specific research field, all of which provide valuable information for researchers involved in the development of research strategies to address various problems. This analysis is based on a statistical and quantitative assessment that allows for an objective evaluation of a given article. It also performs a time-efficient, focused exploration within a larger field.

Bibliometric databases such as Science Citation Index (SCI), Scopus, and Google collect the citations received for each article, allowing a prescreened and targeted article selection based on the impact of the work [1]. This is important because the number of citations is a recognized measurement of the quality of an article [2]. Science Citation Index Expanded (SCI-EXPANDED) is a broad database of almost 10,000 journals with high-impact citations. It covers many fields, including social sciences, humanities, and the arts, and is the most recent journal citation system and database that has been made available by the Web of Science (WOS) [3]. We, and others, have performed numerous bibliometric analyses that address specific disciplines or terms [4,5,6,7,8,9].

Bibliometric analysis becomes especially powerful when applied to long-standing, classic drugs, such as dimethyl fumarate (BG-12, Tecfidera^®^). Dimethyl fumarate was synthesized in 1963. The main pharmacological effects are mediated by DMF itself, and its metabolite, monomethylfumarate. Wipke et al. showed how nuclear factor (erythroid-derived 2)-like 2 (Nrf2)-mediated oxidative stress response pathways were exclusively regulated by DMF, whereas apoptotic pathways were activated by monomethylfumarate [10]. Therefore, Nfr2 activation promotes transcription of genes which have a marked antioxidant function, such as that of hemooxygenase-1, quinone oxidoreductase, glutathione S transferase, and glutathione peroxidase [11,12]. The efficacy and safety of this drug stands out, reducing the rate of relapses per year in patients with multiple sclerosis (MS). The clinical outcomes and safety profile led the Food & Drug Administration (FDA) to approve it for the treatment of MS in 2013. Dimethyl fumarate is metabolized by esterases, yielding monomethylfumarate before reaching systemic circulation. This enzymatic hydrolysis joins other additional metabolic reactions, such as the tricarboxylic acid cycle, but not the cytochrome P450 system [10].

At present, there is no bibliometric study with a global view which analyzes the relevance of publications on dimethyl fumarate. Therefore, the aim of our bibliometric study is to obtain a broad view of the literature on dimethyl fumarate, and to identify the most cited publications—as well as the most relevant features of these publications (authors, journals, and year of publication). The overall goal of this work is to allow the reader to efficiently and properly address the most important and influential articles in this field.

## 2. Results

On 21 September 2020, we conducted an electronic search in the Web of Science database for references that included the term methyl fumarate in the title. This yielded a total of 135 items. Because of the unexpectedly low number of entries for this term, we redefined the search to include the terms BG-12 and Tecfidera. Thus, the final search included dimethyl fumarate, BG-12, or Tecfidera. This second search resulted in a more than 10-fold increase of the number of retrieved citations, up to 1380 items. All of these references accumulated 11,587 citations, with an h-index value of 46, 8.39 average citations per item, and 192.95 average citations per year.

**Figure 1:**
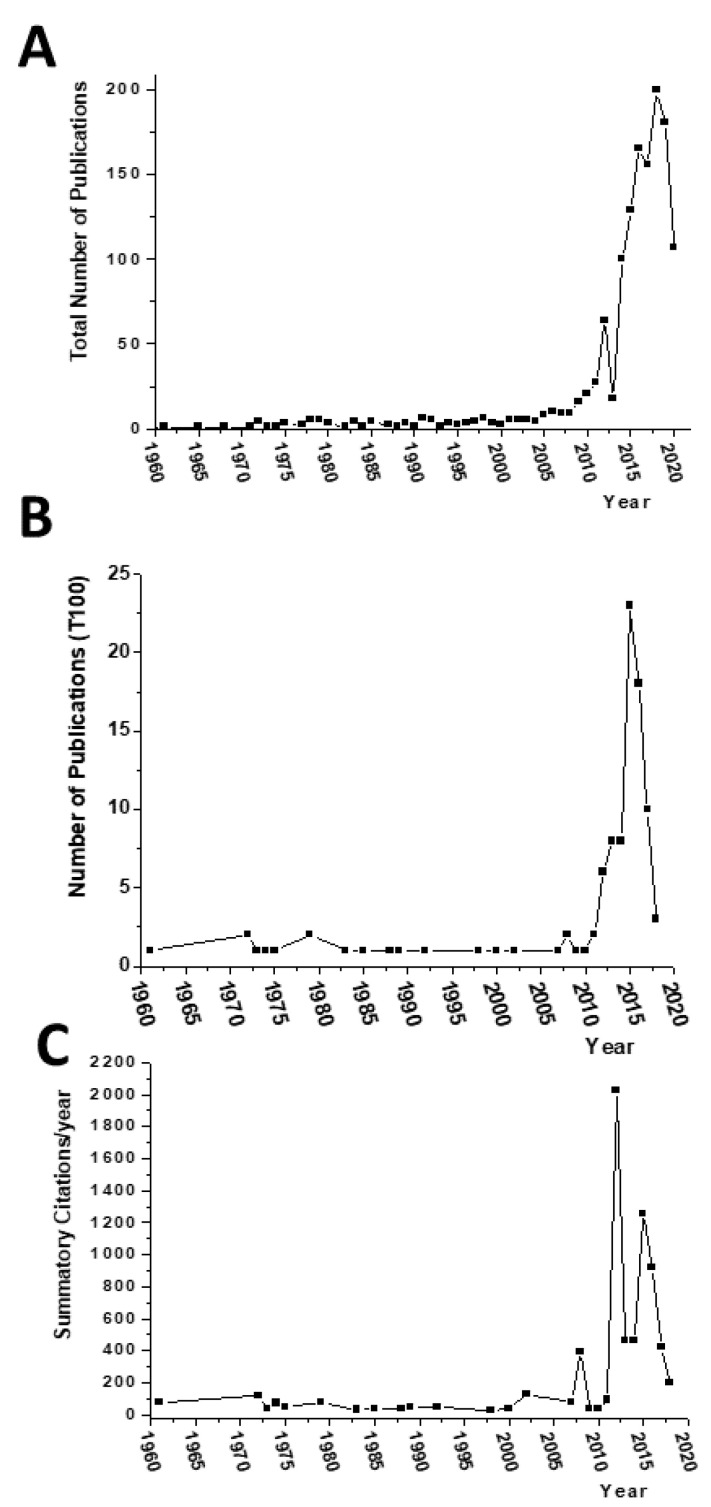
Distribution of the total (**A**), T100 (**B**) articles sorted by the year of publication 10. last years. (**C**) Summatory of the cites T100 per year.

Figure 1A shows the number of publications recorded per year, with the oldest paper dating back to 1950. It can be seen in this figure that, for almost 60 years (1950–2014), the number of published articles on this topic never exceeded ten per year. The year 2014 marked the first time that the number of articles per year exceeded one hundred. Moreover, 75.4% of the total number of papers have been published between 2014 and 2020.

In order to address only original works, we refined our search by filtering for articles, case reports, letters, and clinical trials. This procedure retrieved 677 publications, which we arranged by the total number of citations. The 100 most cited (T100) were then selected. Table 1 depicts the T100, which totaled 11,272 entries from 5350 citing articles, with an h-index of 46 and an aggregate average citation density of 187.87 citations/year. The most cited article (#1) was from the year 2012 (933 citations and a citation density of 103.67 citations/year), while article #100 was published in the year 2017 (28 citations and a citation density of 7 citations/year). The oldest article (from the year 1961) is #19 in the T100 (79 citations; 1.32 citations/year). Papers published in 2018 included three articles which appeared in the T100 ranking: #10, #50, and #92, with 123, 43, and 29 citations, respectively, and a citation density of 41, 14.33, and 9.67 citations/year, respectively.

The T100 ranking list showed an average citation density of 10.01 citations/year. The highest citation density was that of articles #1 and #2 (103.67 and 81.33 citations/year, respectively). The lowest citation density (0.47 citations/year) was article #91.

**Figure 2:**
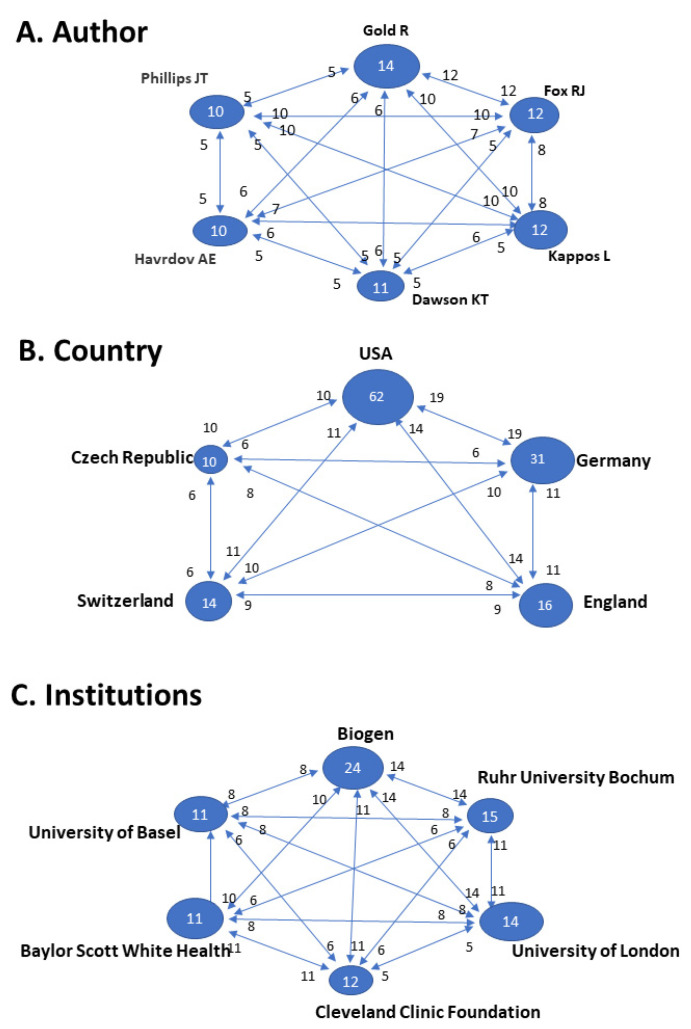
(**A**) The 6 prolific authors within the T100. Individual authors with over 5 publications on the dimethyl fumarate topic sorted by the number of articles published within the T100 list. (**B**) Geographical origin of the T100 Natalizumab publications. Individual country record of articles with more than 10 T100 items sorted by the number of publications Interaction between the above countries. (**C**) Relationship between the institutions with 11 or more publications on topic. Numbers inside the circles indicate the number of articles published. Connecting arrows and numbers affixed indicate number of papers together, respectively.

Next, we focused on the authors of papers in the T100. This analysis was performed independent of the relevance or T100 position. We compiled a total of 478 authors, including affiliations with 145 institutions, across 25 different countries. Table 2 summarizes these findings, showing the five authors with the highest number of publications within the T100. Gold R (Department of Neurology, University of Ruhr, Ruhr, Germany) leads this ranking with 14 articles, accumulating 1823 citations, and an average citation density of 140.23 citations/year (Table 2; Figure 2A).

Figure 2B and Table 3 show the geographic distribution of the T100 list of papers. The highest ranking country was the USA, with 62 articles (5258 citations, 84.81 citations/article and 109.54 citations/year), including 8 of the top 10 (T10). Germany, with 2911 citations (50% lower than the USA), placed second.

According to our study, the most productive institution was Biogen (Cambridge, MA, USA), with 24 items, a total of 3196 citations, and an average citation density of 243.77 citations/year (Table 4). The first T100 item from this institution was published in 2010. Of note, four of the papers from this institution placed in the T10, including the top 3 rankings. Ruhr University Bochum (Germany) had the second-highest number of T100 articles with 15. Figure 2C illustrates the collaborations and interactions between the top 5 institutions, analyzed by detecting coauthorship.

We found 17 research areas publishing articles in the T100, including neurosciences/neurology with 39 articles in the first position, and chemistry with 14 articles (36% of the previous) in the second position. Next, we analyzed whether the percentage of articles in the distribution of research areas changed over time. To do this, we divided the T100 list into two groups of publication, either before or after the year 2000. The 1961–2000 interval included 15 articles, of which 11 (69%) belonged to the chemistry research area and 2 (15%) were found either in the electrochemistry or materials science research areas. No article in this earlier period of time was found within the neurosciences/neurology area. The 2001–2018 interval accounted for the remaining 85 articles, which were included in two main research areas: pharmacology/pharmacy (70 articles, 80%) and neurosciences/neurology (63, articles; 72%). Only 6 papers (6.9%) were within the chemistry area.

The papers ranked in the T100 list were published in 59 journals (Table 5). *Multiple Sclerosis Journal* published 6 articles belonging to this list with 275 citations and an average citation density of 30.56 citations/year. Their first article appearing in T100 was published in 2014. *The New England Journal of Medicine*, with 5 articles in the T100 ranking, is the journal with the highest number of citations (2048, and an average citation density of 277.56 citations/year).

## 3. Discussion

To our knowledge, this is the first bibliometric study of dimethyl fumarate. We believe that this study will help readers to identify the highest-quality papers with the most relevant discoveries and trends regarding this drug’s history and evolution. This study offers three main relevant conclusions. The first is that any electronic search for dimethyl fumarate should also include the terms BG-12 and Tecfidera. The second is that, when we consider the total number of articles published per year, our results show a sharp point of inflection in the year 2013. One of the strengths of this study is that it has been performed without a date limit, elucidating the fluctuations in interest in this drug. This led us to the third major point: the clear change after 2013 in the areas of research covering this topic. Before 2013, the leading research area was chemistry and after 2013 it was neurosciences/neurology.

When we studied the T10 publications, we found three groups of papers: (i) clinical trials, (ii) papers reporting side effects, and (iii) papers focused on the mechanism of action of the drug. The three most cited articles were clinical trials. Of these, both #1 and #2 compared the effects of 240 mg of dimethyl fumarate administered twice or three times a day versus a placebo. A particular point of interest for citations of paper #1 is that the authors included data on the effects of dimethyl fumarate on incapacity progression. While paper #2 additionally reported on the effects of glatiramer acetate, the number of citations was lower. This is possibly due to the fact that this clinical trial was not designed to investigate the differences between the two drugs. Despite being older than papers #1 and 2, publication #3 was cited less, probably because it was a phase IIb trial with a small number of patients. Articles #5 and #6 reported cases of side effects of dimethyl fumarate in which patients developed progressive multifocal leukoencephalopathy. Articles #4 and #10 studied the anti-inflammatory mechanism of action. It is worth mentioning that they were carried out after clinical trials were published demonstrating the efficacy of dimethyl fumarate in MS. Both articles highlight that the mechanism of action is not mediated, as was previously thought, through the action of dimethyl fumarate on the activation of the nuclear factor (erythroid-derived 2)-like 2 (Nrf2) antioxidative pathway. Most interesting is that these articles suggested possible pharmacological targets in the development of anti-MS drugs such as hydroxycarboxylic acid receptor 2 or the downregulation of aerobic glycolysis.

Our results are consistent with Bradford’s Law, which establishes that most articles of interest in an area are published in a small number of high-impact journals [13]. All T10 articles have been published in top quartile (Q1) journals of their respective areas of knowledge, including four in the *New England Journal of Medicine*. Furthermore, we found that the highest correlations obtained were between the number of citations and the journal impact factor or with the article influence score. Interestingly, both correlated more than with the Eigenfactor, probably because of the way this factor is calculated (the Eigenfactor rates the citations obtained from higher ranked journals more highly) which highlights different citation patterns within each discipline and eliminates self-care [14].

With regard to institutions published within the T100, Biogen stood out with a total of 24 articles. Furthermore, Biogen supported at least three articles within the T10 (#1, #2, and #9), all of which provided helpful data on the efficacy of dimethyl fumarate. Not surprisingly, it is this institution that launched the drug in 2013 [15]. Biogen was also the institution with the highest number of articles within the T100 in a previous bibliometric study conducted by our research group and centered on natalizumab, a humanized anti-alpha-4 integrin antibody used in the management of MS [4].

If we focus on the countries where the research publication took place, the USA was #1, with 62 articles and 5258 citations. This was followed by Germany, with 31 articles and 2911 citations.

It cannot be ignored that the present work, like other bibliometric studies, has limitations. We would like to address two of them. The first is that this bibliometric study could be labeled as a mere snapshot taken on a certain date. While this is true, it can be counterweighted by taking into account the density of citations per year for each particular publication. Using this parameter, we should be able to determine whether or not a specific article maintains the interest of the scientific community. It is also possible to anticipate if a specific article’s position within the T100 will change. For instance, we can predict that article #10 of the T100, which has an average citation density of 41.0 citations/year should rise in its ranking within the T100, reaching the top 4. Interestingly, article #10 addresses a possible mechanism of action of dimethyl fumarate at an immunomodulatory level, where it would inhibit aerobic glycolysis in myeloid and lymphoid cells, causing an anti-inflammatory effect which might be useful in immune diseases such as MS and psoriasis. 

Another study limitation is that this search was performed using a single bibliometric database. However, it is important to stress that we chose this database because we aimed to study the drug’s history and evolution over time. Other bibliometric databases, including Scopus, reference only recent citations.

In conclusion, we have tried to shed light on the use of dimethyl fumarate over the years by searching for information on which publications are the most cited, as well as the associated investigators, countries, and institutions participating in the studies. We highlighted changes in the leading research areas that have taken place since this drug was first synthesized. This will provide readers with a strong tool to efficiently select the most relevant articles on dimethyl fumarate for their own interest, research and clinical applications.

## 4. Material and Methods

On 21 September 2020 we carried out the last literature search on the Web of Science (WOS, Clarivate Analytics, Philadelphia, PA, USA) of all articles containing the terms dimethyl fumarate, BG-12 or Tecfidera. The databases used were: SCI-EXPANDED, Social Science Citation Index (SSCI), Arts & Humanities Citation Index (A & HCI), Conference Proceedings Citation Index-Science (CPCI-S), Conference Proceedings Citation Index-Social Science & Humanities (CPCI-SSH), Emerging Sources Citation Index (ESCI), Current Chemical Reactions-Expanded (CCR-EXPANDED), and Index Chemicus (IC). No filters were applied to the period of publication, language of publication, authors, participating institutions, topics, or grant funding of the articles. We would like to remark that this project required several searches and studies before the present analysis was achieved. As a result of these searches, we grew to understand the bibliometric behavior of this drug. For instance, we realized that the search should include the terms BG-12 and Tecfidera in addition to dimethyl fumarate. After performing preliminary searches, we also came to the decision that the final analysis should be limited to entries which include the name of the drug as part of the title of the article, excluding those where it appears only in the abstract. Then, in order to obtain only original works, we refined the search to article, early access, other, case report, and clinical trial.

Articles were sorted by the number of citations, and we selected the one hundred most cited (T100). In these articles, we proceeded to study the following variables: author, title, number of times cited, source, identification of authors, institution or particular country in which a T100 article has been published, citation density (citations/year), and citations per record. The obtained data were exported to an Excel spreadsheet (Microsoft, Redmond, WA, USA). We defined a “classic” as a research work that has been cited at least 100 times. An estimate of the number of citations expected within five years was made by taking the number of total citations and adding five times the number of annual citations from this same article. In addition, journal name and impact factor (IF) were also extracted. Journal IF’s were cross-referenced with the 2020 edition of the Journal Citation Reports (JCR): Science Edition (1945–2020). The calculation of Pearson’s correlation coefficient between total citations, annual citations, and the estimate of citations within five years was carried out according to the impact factor of the journal in 2019, the Eigenfactor, and the Article Influence score. A value of *p* < 0.05 is considered statistically significant. For the statistical analysis, SPSS (version 21.0 IBM Co. Armonk, NY, USA) was used. None of the authors of this manuscript are related to any of the pharmaceutical industries involved in the research or production of this drug.

## Figures and Tables

**Table 1:** Bibliometric information associated with the top 100 (T100) cited articles.

Rank #	Article Title	Journal	Year	Times Quoted	No. of Citations Per Year
1	Placebo-Controlled Phase 3 Study of Oral BG-12 for Relapsing Multiple Sclerosis	*New England Journal of Medicine*	2012	933	103.67
2	Placebo-Controlled Phase 3 Study of Oral BG-12 or Glatiramer in Multiple Sclerosis	*New England Journal of Medicine*	2012	732	81.33
3	Efficacy and safety of oral fumarate in patients with relapsing-remitting multiple sclerosis: a multicentre. Randomised. Double-blind. Placebo-controlled phase IIb study	*Lancet*	2008	360	27.69
4	Hydroxycarboxylic acid receptor 2 mediates dimethyl fumarate’s protective effect in EAE	*Journal of Clinical Investigation*	2014	148	21.14
5	PML in a Patient Treated with Dimethyl Fumarate from a Compounding Pharmacy	*New England Journal of Medicine*	2013	146	18.25
6	PML in a Patient with Lymphocytopenia Treated with Dimethyl Fumarate	*New England Journal of Medicine*	2015	133	22.17
7	Growth of Campylobacter jejuni supported by respiration of fumarate. Nitrate. Nitrite. Trimethylamine-N-oxide. Or dimethyl sulfoxide requires oxygen	*Journal of Bacteriology*	2002	128	6.74
8	Dimethyl fumarate in the treatment of relapsing-remitting multiple sclerosis: an overview	*Therapeutic Advances in Neurological Disorders*	2015	124	20.67
9	Effects of dimethyl fumarate on neuroprotection and immunomodulation	*Journal of Neuroinflammation*	2012	124	13.78
10	Dimethyl fumarate targets GAPDH and aerobic glycolysis to modulate immunity	*Science*	2018	123	41
11	Dimethyl Fumarate Inhibits Dendritic Cell Maturation via Nuclear Factor kappa B (NF-kappa B) and Extracellular Signal-regulated Kinase 1 and 2 (ERK1/2) and Mitogen Stress-activated Kinase 1 (MSK1) Signaling	*Journal of Biological Chemistry*	2012	120	13.33
12	Dimethyl fumarate treatment induces adaptive and innate immune modulation independent of Nrf2	*Proceedings of The National Academy of Sciences of The United States of America*	2016	119	23.8
13	Reduction of CD8(+) T lymphocytes in multiple sclerosis patients treated with dimethyl fumarate	*Neurology-Neuroimmunology & Neuroinflammation*	2015	111	18.5
14	The anti-inflammatory effects of dimethyl fumarate in astrocytes involve glutathione and haem oxygenase-1	*ASN Neuro*	2011	109	10.9
15	PML in a Patient without Severe Lymphocytopenia Receiving Dimethyl Fumarate	*New England Journal of Medicine*	2015	104	17.33
16	Repurposing the NRF2 Activator Dimethyl Fumarate as Therapy Against Synucleinopathy in Parkinson’s Disease	*Antioxidants & Redox Signaling*	2016	100	20
17	Electrohydrodimerization Reactions. 2. Rotating Ring-Disk Electrode. Voltammetric and Coulometric Studies of Dimethyl Fumarate. Cinnamonitrile. and Fumaronitrile	*Journal of The Electrochemical Society*	1972	87	1.78
18	Reactivity of dimethyl fumarate and methylhydrogen fumarate towards glutathione and N-acetyl-L-cysteine—Preparation of S-substituted thiosuccinic acid esters	*Bioorganic & Medicinal Chemistry*	2007	82	5.86
19	Chemistry of Photodimers of Maleic And Fumaric Acid Derivatives. 1. Dimethyl Fumarate Dimer	*Journal of The American Chemical Society*	1961	79	1.32
20	Dimethyl fumarate treatment alters circulating T helper cell subsets in multiple sclerosis	*Neurology-Neuroimmunology & Neuroinflammation*	2016	76	15.2
21	Dimethyl Fumarate and Monoethyl Fumarate Exhibit Differential Effects on KEAP1. NRF2 Activation. And Glutathione Depletion In Vitro	*Plos One*	2015	73	12.17
22	Dimethyl Fumarate for Treatment of Multiple Sclerosis: mechanism of Action, effectiveness and side effects	*Current Neurology And Neuroscience Reports*	2013	73	9.13
23	Role of a singlet exciplex in photocycloaddition of phenanthrene to dimethyl fumarate	*Journal of The American Chemical Society*	1974	72	1.53
24	BG-12 (dimethyl fumarate): a review of mechanism of action efficacy and safety	*Current Medical Research and Opinion*	2014	71	10.14
25	Dimethyl fumarate protects neural stem/progenitor cells and neurons from oxidative damage through Nrf2-ERK1/2 MAPK Pathway	*International Journal of Molecular Sciences*	2015	67	11.17
26	Chemical proteomic map of dimethyl fumarate-sensitive cysteines in primary human T cells	*Science Signaling*	2016	66	13.2
27	Dimethyl fumarate selectively reduces memory t cells and shifts the balance between Th1/Th17 and Th2 in multiple sclerosis patients	*Journal of Immunology*	2017	64	16
28	Long-term effects of delayed-release dimethyl fumarate in multiple sclerosis: Interim analysis of ENDORSE. A randomized extension study	*Multiple Sclerosis Journal*	2017	64	16
29	Dimethyl fumarate selectively reduces memory T cells in multiple sclerosis patients	*Multiple Sclerosis Journal*	2016	63	12.6
30	Dimethyl fumarate—only an anti-psoriatic medication?	*Journal Der Deutschen Dermatologischen Gesellschaft*	2012	63	7
31	Dimethyl fumarate induces necroptosis in colon cancer cells through GSH depletion/ROS increase/MAPKs activation pathway	*British Journal of Pharmacology*	2015	62	10.33
32	Dimethyl fumarate treatment mediates an anti-inflammatory shift in b cell subsets of patients with multiple sclerosis	*Journal of Immunology*	2017	58	14.5
33	Effect of BG-12 on contrast-enhanced lesions in patients with relapsing-remitting multiple sclerosis: subgroup analyses from the phase 2b study	*Multiple Sclerosis Journal*	2012	51	5.67
34	Dimethyl fumarate ameliorates dextran sulfate sodium-induced murine experimental colitis by activating Nrf2 and suppressing NLRP3 inflammasome activation	*Biochemical Pharmacology*	2016	50	10
35	Dimethyl fumarate protects brain from damage produced by intracerebral hemorrhage by mechanism involving Nrf2	*Stroke*	2015	50	8.33
36	Dimethyl fumarate. An immune modulator and inducer of the antioxidant response. Suppresses HIV replication and macrophage-mediated neurotoxicity: a novel candidate for HIV neuroprotection	*Journal of Immunology*	2011	50	5
37	Effects of delayed-release dimethyl fumarate on MRI measures in the Phase 3 DEFINE study	*Journal of Neurology*	2014	49	7
38	BG-12 in Multiple Sclerosis	*Seminars In Neurology*	2013	48	6
39	1.3-Cycloadditions of aliphatic thione s-methylides to dimethyl 2.3-dicyano-fumarate and 2.3-dicyanomaleate—a test case for steric course and mechanism	*Tetrahedron Letters*	1989	48	1.5
40	Dimethyl fumarate attenuates 6-OHDA-induced neurotoxicity in sh-sy5y cells and in animal model of Parkinson’s disease by enhancing Nrf2 activity	*Neuroscience*	2015	47	7.83
41	Efficacy and safety of BG-12 (dimethyl fumarate) and other disease-modifying therapies for the treatment of relapsing-remitting multiple sclerosis: a systematic review and mixed treatment comparison	*Current Medical Research and Opinion*	2014	47	6.71
42	Clinical efficacy of BG-12 (dimethyl fumarate) in patients with relapsing-remitting multiple sclerosis: subgroup analyses of the DEFINE study	*Journal of Neurology*	2013	47	5.88
43	Kinetic-studies on the radical polymerization of isopropyl tert-butyl fumarate initiated with 2.2′-azobis (isobutyronitrile) and dimethyl 2.2′-azobis(isobutyrate)—rates of addition and termination of the primary radicals	*Macromolecules*	1992	47	1.62
44	Singlet and triplet exciplexes in photoreaction of phenanthrene with dimethyl fumarate	*Journal of The American Chemical Society*	1975	47	1.02
45	Dimethyl fumarate treatment of relapsing-remitting multiple sclerosis influences B-cell subsets	*Neurology-Neuroimmunology & Neuroinflammation*	2016	46	9.2
46	Dimethyl fumarate in relapsing-remitting multiple sclerosis: rationale. Mechanisms of action. Pharmacokinetics. Efficacy and safety	*Expert Review of Neurotherapeutics*	2015	46	7.67
47	Clinical efficacy of BG-12 (dimethyl fumarate) in patients with relapsing-remitting multiple sclerosis: subgroup analyses of the CONFIRM study	*Journal of Neurology*	2013	46	5.75
48	Dimethyl fumarate inhibits the Nuclear Factor B pathway in breast cancer cells by covalent modification of p65 protein	*Journal of Biological Chemistry*	2016	45	9
49	Dimethyl fumarate attenuates cerebral edema formation by protecting the blood-brain barrier integrity	*Experimental Neurology*	2015	44	7.33
50	Emerging understanding of the mechanism of action for dimethyl fumarate in the treatment of multiple sclerosis	*Frontiers In Neurology*	2018	43	14.33
51	Dimethyl fumarate and monomethyl fumarate promote post-ischemic recovery in mice	*Translational Stroke Research*	2016	43	8.6
52	BG-12 reduces evolution of new enhancing lesions to T1-hypointense lesions in patients with multiple sclerosis	*Journal of Neurology*	2011	43	4.3
53	[2 + 2] cycloadditions of silyl enol ethers and dimethyl acetylenedicarboxylate. Dimethyl fumarate and methyl crotonate	*Journal of Organic Chemistry*	1979	43	1.02
54	Dimethyl fumarate blocks proinflammatory cytokine production via inhibition of TLR induced M1 and K63 ubiquitin chain formation	*Scientific Reports*	2016	42	8.4
55	Dimethyl fumarate: a review of its use in patients with relapsing-remitting multiple sclerosis	*CNS Drugs*	2014	42	6
56	Dimethyl fumarate in multiple sclerosis: latest developments. Evidence and place in therapy	*Therapeutic Advances In Chronic Disease*	2016	41	8.2
57	Dimethyl fumarate modulation of immune and antioxidant responses: application to HIV therapy	*Critical Reviews In Immunology*	2013	41	5.13
58	Characterizing absolute lymphocyte count profiles in dimethyl fumarate-treated patients with MS Patient management considerations	*Neurology-Clinical Practice*	2016	40	8
59	Cyclo-additions of N-aryl-C-(trifluoromethyl)nitrilimines with dimethyl fumarate and maleate	*Journal of Heterocyclic Chemistry*	1985	40	1.11
60	Dimethyl fumarate-induced lymphopenia in MS due to differential T-cell subset apoptosis	*Neurology-Neuroimmunology & Neuroinflammation*	2017	39	9.75
61	The effect of dimethyl fumarate (Tecfidera (TM)) on lymphocyte counts: A potential contributor to progressive multifocal leukoencephalopathy risk	*Multiple Sclerosis And Related Disorders*	2015	39	6.5
62	The neuroprotective effect of dimethyl fumarate in an MPTP-mouse model of parkinson’s disease: involvement of reactive oxygen species/Nuclear Factor-kappa B/nuclear transcription factor related to NF-e2	*Antioxidants & Redox Signaling*	2017	38	9.5
63	Efficacy and safety of LAS41008 (dimethyl fumarate) in adults with moderate-to-severe chronic plaque psoriasis: a randomized. Double-blind. Fumaderm (R)—and placebo-controlled trial (BRIDGE)	*British Journal of Dermatology*	2017	38	9.5
64	Dimethyl fumarate confers neuroprotection by casein kinase 2 phosphorylation of Nrf2 in murine intracerebral hemorrhage	*Neurobiology of Disease*	2015	38	6.33
65	Effect of dimethyl fumarate on the radiation sensitivity of mammalian-cells invitro	*Radiation Research*	1988	38	1.15
66	Utilization of dimethyl fumarate and related molecules for treatment of multiple sclerosis cancer and other diseases	*Frontiers In Immunology*	2016	37	7.4
67	Effects of delayed-release dimethyl fumarate on mri measures in the phase 3 CONFIRM study	*Neurology*	2015	37	6.17
68	Shoe contact dermatitis from dimethyl fumarate: clinical manifestations. Patch test results. Chemical analysis. And source of exposure	*Contact Dermatitis*	2009	37	3.08
69	Electrohydrodimerization reactions. 3. Rotating-ring-disk electrode. Voltammetric and coulometric studies of mixed reductive coupling of dimethyl fumarate in presence of cinnamonitrile and acrylonitrile in dimethylformamide solution	*Journal of The Electrochemical Society*	1973	37	0.77
70	Progressive neurologic dysfunction in a psoriasis patient treated with dimethyl fumarate	*Annals of Neurology*	2015	36	6
71	Quality of life outcomes with BG-12 (dimethyl fumarate) in patients with relapsing-remitting multiple sclerosis: The DEFINE study	*Multiple Sclerosis Journal*	2014	36	5.14
72	Dimethyl fumarate for multiple sclerosis	*Expert Opinion On Investigational Drugs*	2010	36	3.27
73	Dimethyl fumarate modulates antioxidant and lipid metabolism in oligodendrocytes	*Redox Biology*	2015	35	5.83
74	Activation of Nrf2 by dimethyl fumarate improves vascular calcification	*Vascular Pharmacology*	2014	35	5
75	Kinetics of 1.3-dipolar cycloaddition reaction between C.N-diphenylnitrone and dimethyl fumarate in various solvents and aqueous solutions	*International Journal of Chemical Kinetics*	2000	35	1.67
76	Control of oxidative stress and inflammation in sickle cell disease with the Nrf2 activator dimethyl fumarate	*Antioxidants & Redox Signaling*	2017	34	8.5
77	Dimethyl fumarate attenuates experimental autoimmune neuritis through the nuclear factor erythroid-derived 2-related factor 2/hemoxygenase-1 pathway by altering the balance of M1/M2 macrophages	*Journal of Neuroinflammation*	2016	34	6.8
78	Dimethyl fumarate. A small molecule drug for psoriasis. Inhibits Nuclear Factor-kappa B and reduces myocardial infarct size in rats	*European Journal of Pharmacology*	2008	34	2.62
79	Efficacy of delayed-release dimethyl fumarate in relapsing-remitting multiple sclerosis: integrated analysis of the phase 3 trials	*Annals of Clinical and Translational Neurology*	2015	33	5.5
80	Efficacy and safety of delayed-release dimethyl fumarate in patients newly diagnosed with relapsing-remitting multiple sclerosis (RRMS)	*Multiple Sclerosis Journal*	2015	33	5.5
81	Tolerability and pharmacokinetics of delayed-release dimethyl fumarate administered with and without aspirin in healthy volunteers	*Clinical Therapeutics*	2013	33	4.13
82	Synthesis of 3-co-ordinate mono-olefin. Bis-olefin and tris-olefin complexes of platinum with dimethyl or diethyl fumarate, imethyl maleate or maleic-anhydride ligands	*Journal of The Chemical Society-Dalton Transactions*	1979	33	0.79
83	Dimethyl fumarate and the oleanane triterpenoids. Cddo-imidazolide and cddo-methyl ester. Both activate the Nrf2 pathway but have opposite effects in the A/J model of lung carcinogenesis	*Carcinogenesis*	2015	32	5.33
84	Preparation of dimethyl 2-(phenylthio)maleate Dimethyl 2-(phenylthio)fumarate and their sulfoxides	*Journal of Organic Chemistry*	1983	32	0.84
85	Dimethyl fumarate alters B-cell memory and cytokine production in MS patients	*Annals of Clinical and Translational Neurology*	2017	31	7.75
86	PML during dimethyl fumarate treatment of multiple sclerosis: How does lymphopenia matter?	*Neurology*	2016	31	6.2
87	Delayed-release dimethyl fumarate and pregnancy: preclinical studies and pregnancy outcomes from clinical trials and postmarketing experience	*Neurology and Therapy*	2015	31	5.17
88	Pharmacology and clinical efficacy of dimethyl fumarate (BG-12) for treatment of relapsing-remitting multiple sclerosis	*Therapeutics and Clinical Risk Management*	2014	31	4.43
89	Effect of delayed-release dimethyl fumarate on no evidence of disease activity in relapsing-remitting multiple sclerosis: integrated analysis of the phase III DEFINE and CONFIRM studies	*European Journal of Neurology*	2017	30	7.5
90	Nonfatal PML in a patient with multiple sclerosis treated with dimethyl fumarate	*Neurology-Neuroimmunology & Neuroinflammation*	2016	30	6
91	Reactions of exciplex from singlet-excited phenanthrene and dimethyl fumarate-oxetan formation. Intersystem crossing and emission	*Journal of The Chemical Society-Chemical Communications*	1972	30	0.61
92	Dimethyl fumarate influences innate and adaptive immunity in multiple sclerosis	*Journal of Autoimmunity*	2018	29	9.67
93	Dimethyl fumarate restores apoptosis sensitivity and inhibits tumor growth and metastasis in CTCL by targeting F-kappa B	*Blood*	2016	29	5.8
94	Pharmacodynamics of dimethyl fumarate are tissue specific and involve Nrf2-dependent and -independent mechanisms	*Antioxidants & Redox Signaling*	2016	29	5.8
95	Effects of dimethyl fumarate on lymphocyte subsets	*Multiple Sclerosis and Related Disorders*	2015	29	4.83
96	Solubility of dimethyl fumarate in water plus (methanol. Ethanol. 1-propanol) from (278.15 to 333.15) K	*Fluid Phase Equilibria*	2013	29	3.63
97	Synthesis of (3S.4R)-3.4-isopropylidenedioxy-1-pyrroline-N-oxide. An enantiopure functionalized cyclic nitrone; Cycloaddition reactions with dimethyl maleate and dimethyl fumarate	*Synthetic Communications*	1998	29	1.26
98	Dimethyl fumarate mediates Nrf2-dependent mitochondrial biogenesis in mice and humans	*Human Molecular Genetics*	2017	28	7
99	Dimethyl fumarate associated lymphopenia in clinical practice	*Multiple Sclerosis Journal*	2015	28	4.67
100	Dimethyl fumarate for multiple sclerosis	*Cochrane Database of Systematic Reviews*	2015	28	4.67

**Table 2:** Authors with 11 or more T100 articles. Authors are arranged by the number of T100 articles in his/her curriculum (T100-record).

Author	Affiliation	T100-Record	Times Cited	Average Citations/Record	Average Citations/Year
Gold R	Department of Neurology. Perelman School of Medicine, University of Pennsylvania; USA	12	1823	130.21	140.23
Fox RJ	Mellen Center for Multiple Sclerosis; Neurological Institute. Cleveland. USA	12	1211	100.92	134.56
Kappos L	St. Josef Hospital, Department of Neurology Ruhr University, Bochum, Germany.	12	1709	142.42	131.46
Dawson KT	Biogen Idec, Inc. Cambridge, Massachusetts USA	11	2377	218.09	182.5
Phillips JT	Baylor Institute for Immunology Research Dallas, Texas, USA	10	1101	110.10	122.33

**Table 3:** Countries publishing more than 10 of the T100 cited articles.

Country	T100-Record	Times Cited	Average Citations/Record	Average Citations/Year
USA	62	5258	109.54	84.81
Germany	30	2911	207.93	97.03
England	16	2655	63.21	165.94
Switzerland	14	1862	143.23	133
Czech Republic	10	1440	110.77	144

**Table 4:** Institutions publishing more than 10 of the T100 manuscript are arranged by the number of T100 records.

Institutions	Country	T100-Record	Times Cited	Average Citations/Record	Average Citation Density
Biogen	USA	24	3169	132.04	243.77
Ruhr University Bochum	Germany	15	1864	124.77	143.38
University of London	England	14	2494	178.14	191.85
Cleveland Clinic Foundation	USA	12	1211	100.92	134.65
Baylor Scott White Health	USA	11	1165	105.91	129.44
University of Basel	Switzerland	11	1682	152.91	129.38

**Table 5:** Journals publishing more than 4 of the top 100 (T100) papers are arranged by the number of T100 records.

Journal	T100-Record	Impact Factor	Eigenfactor	Article Influence	Times Cited	Average Citations/Record	Citation Density
*Neurology Neuroimmunology Neuroinflammation*	5	8.27	0.021	2.4	302	60.4	50.33
*New England Journal of Medicine*	5	74.70	0.682	25.7	2048	409.6	227.56
*Antioxidants Redox Signaling*	4	60.39	-	-	201	50.25	40.20
*Journal of Neurology*	4	2.98	-	-	185	46.25	18.50
